# The transformation of grid to place cells is robust to noise in the grid pattern

**DOI:** 10.1186/1471-2202-15-S1-P188

**Published:** 2014-07-21

**Authors:** Amir H Azizi, Sen Cheng

**Affiliations:** 1 Department of Psychology, Ruhr-University Bochum, Bochum, NRW, 44801, Germany

## 

The neural mechanisms of spatial navigation in rodents are thought to rest on place-selective cells in the hippocampus and/ or medial entorhinal cortex (MEC). These cells have been suggested to be the basis for a cognitive map of the animal's environment and for path integration. However, despite large body of theoretical work, it remains unsettled how the neural responses of hippocampal place cells and the grid cells in MEC are generated. Given the massive projections from the superficial MEC to the hippocampal CA regions, which host the place cells, it was initially postulated that grid cells drive the spatial responses of place cells. The transformation was modeled as a linear weighted summation of input grid cells into place cells firing. Different strategies was used to implement the connecting weights (see [1] for a generalized model and review of earlier models).

**Figure 1:**
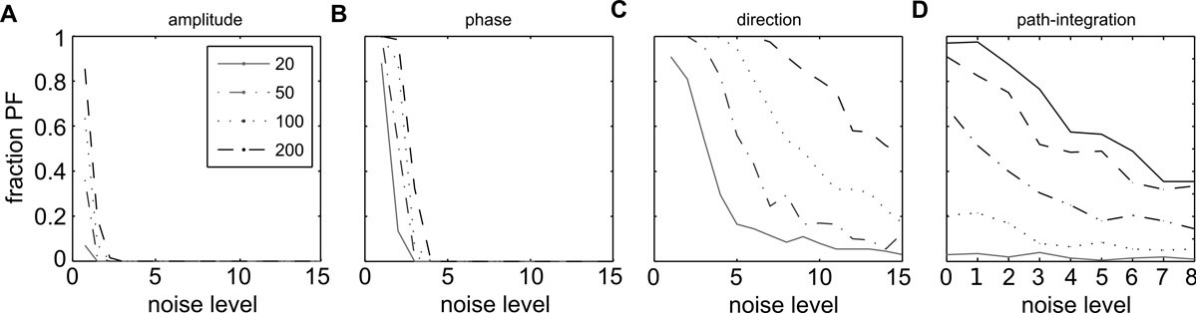
Robustness of grid to place transformation under different noise strategies. Fraction of simulation runs that lead to stable place cell as a function of added noise to the amplitude (**A**), phase (**B**), direction (**C**) of the grid pattern and accumulated noise due to path-integration error (**D**). For large grid cell networks, the transformation is robust to the addition of noise to the direction of unit vectors of the corresponding grid cells and the accumulated error due to path-integration.

However, recent experimental evidence appears to cast doubt on this suggestion. During development [2] or, when the theta oscillation was disrupted due to medial septum inactivation [3], stable place cell responses were found at the same time when the grid cell responses were severely distorted. The authors of these studies therefore concluded that place cells cannot rely on grid cells as their exclusive source of input. In this work, we use computational modeling to show that this conclusion might have been premature. We use a simple feedforward network with grid cells in one layer and place cells in another layer to model the grid-to-place cell transformation. The weights in this network depend on the spatial phase of the grid cell according to a relationship that we reported previously [1]. We perturb the grids in the input in four different ways and study the robustness of the place cells response to these perturbations. In two cases, which we do not regard as realistic, we find that the grid-to-place cell transformation is not robust (Figure 1 A and B). However, in the more realistic two cases of grid perturbations, the transformation is very robust (Figure 1 C and D).

These robust cases include grid perturbations due to noise in the path integration mechanism that gives rise to grid cell responses and noise in the alignment of the three main axes of the grids. Although these two cases are conceptually quite different, many results are quite similar. Even if current experimental data suggest the involvement of other inputs in driving place cell responses, our work shows that the simple idea that grid cells drive spatial responses of place cells cannot be ruled out at this point.
